# Anterior cervical discectomy and fusion: Comparison of titanium and polyetheretherketone cages

**DOI:** 10.1186/1471-2474-13-172

**Published:** 2012-09-14

**Authors:** Mario Cabraja, Soner Oezdemir, Daniel Koeppen, Stefan Kroppenstedt

**Affiliations:** 1 Department of Neurosurgery, Charité-Universitätsmedizin Berlin, Hindenburgdamm 20, Berlin, 12200, Germany

## Abstract

**Background:**

Titanium (TTN) cages have a higher modulus of elasticity when compared with polyetheretherketone (PEEK) cages. This suggests that TTN-cages could show more frequent cage subsidence after anterior cervical discectomy and fusion (ACDF) and therefore might lead to a higher loss of correction. We compared the long term results of stand-alone PEEK- and TTN-cages in a comparable patient collective that was operated under identical operative settings.

**Methods:**

From 2002 to 2007 154 patients underwent single-level ACDF for degenerative disc disease (DDD). Clinical and radiological outcome were assessed in 86 eligible patients after a mean of 28.4 months. 44 patients received a TTN- and 42 patients a PEEK-cage.

**Results:**

Solid arthrodesis was found in 93.2% of the TTN-group and 88.1% of the PEEK-group. Cage subsidence was observed in 20.5% of the TTN- and 14.3% of the PEEK-group. A significant segmental lordotic correction was achieved by both cage-types. Even though a loss of correction was found at the last follow-up in both groups, it did not reach the level of statistical significance. Statistical analysis of these results revealed no differences between the TTN- and PEEK-group.

When assessed with the neck disability index (NDI), the visual analogue scale (VAS) of neck and arm pain and Odom’s criteria the clinical data showed no significant differences between the groups.

**Conclusions:**

Clinical and radiological outcomes of ACDF with TTN- or PEEK-cages do not appear to be influenced by the chosen synthetic graft. The modulus of elasticity represents only one of many physical properties of a cage. Design, shape, size, surface architecture of a cage as well as bone density, endplate preparation and applied distraction during surgery need to be considered as further important factors.

## Background

Cervical titanium (TTN) and polyetheretherketone (PEEK) cages for intervertebral disc space reconstruction are both accepted grafts for anterior cervical discectomy and fusion (ACDF) [1-7].

TTN-cages have been criticized to produce an inferior clinical outcome compared with bone grafts due to a higher elasticity modulus, which could result in subsidence [8]. Nevertheless, due to structural properties TTN implants are likely to provide a good osseointegration [9] and several clinical studies demonstrate successful results after implantation of TTN-cages [10-13].

PEEK-cages have a modulus of elasticity closely resembling that of cortical bone, which might lead to advantages in load sharing and stress distribution. This might result in a lower subsidence rate with less loss of segmental correction and potentially higher fusion rate [14-16].

A direct comparison of cervical TTN- and PEEK-cages in a clinical setting is very rarely found in the literature [16-19], and even less studies consequently compare the radiological results [16,18]. The latter studies showed the PEEK-implants being superior in maintaining cervical interspace height and achieving radiographic fusion[16,18], even suggesting to cease the application of TTN-cages in cervical spine surgery [16].

The aim of the present study was to evaluate the fusion of the operated segment and to examine the rate of cage subsidence as well as clinical outcome in two comparable patient collectives.

## Methods

### Patient cohort

**Figure 1:**
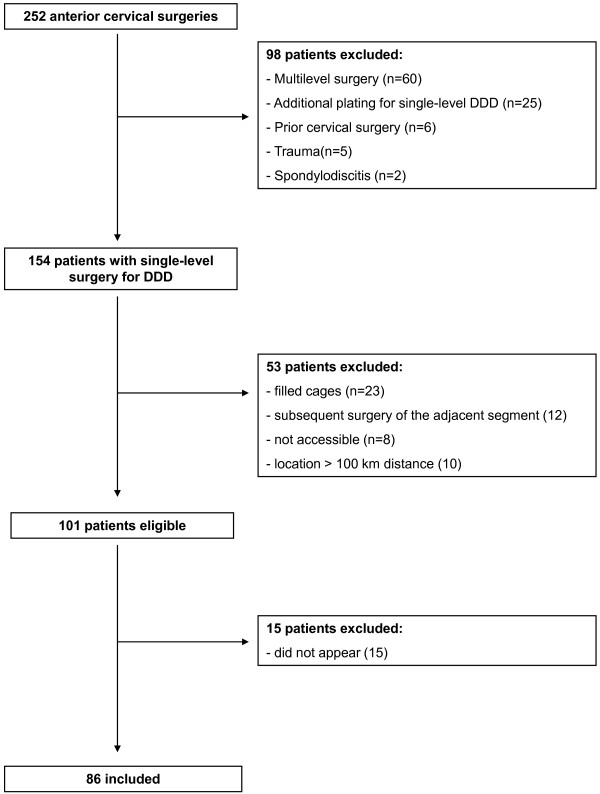
Patient flow.

From 2002 to 2007 according to our records a total of 252 patients underwent ACDF. For better comparison we have excluded patients with multilevel surgery, previous or subsequent surgery of the cervical spine, cage fillings with allo-, autograft or bone substitutes, additional plating for single-level ACDF or patients suffering from an infection or traumatic spinal cord injury. Patients that did not appear to at least one-year follow-up were excluded as well (see Figure 1).

The study conforms to the Helsinki Declaration, and to local legislation. By protecting the patients’ anonymity, approval of our institutional ethics committee is not required for this retrospective study.

The included patients experienced radiculopathy and neck pain as main symptoms. 16 patients suffered from a myelopathy (see Table 1). All of the patients underwent conservative treatment unsuccessfully.

**Table 1:** Demographic and clinical data of the patients

**Variable**	**TTN (n = 44)**	**PEEK (n = 42)**	**p-value**
**Age at surgery (years)**	51.09 ± 8.88	57.64 ± 11.10	0.030
**Gender**			0.768
Male	26	28	
Female	18	14	
**Smoker**			0.400
Yes	27	22	
No	17	20	
**Follow-up (months)**	30.568 ± 14.32	26.1 ± 9.97	0.096
**Radiculopathy**	36	34	0.796
**Myelopathy**	8	8	0.918

### Surgery

ACDF was performed in supine position by a transverse skin incision from the right side after induction of general anaesthesia. After removal of the disc, preparation of the endplates with a rongeur and decompression of the nerval structures the intervertebral space was filled with an empty stand-alone synthetic cage under anterior distraction. Great care was taken to remove the cartilaginous tissue, but preserve intact endplates. No drill was used for the preparation of the endplates. The cage was placed close to the anterior margin of the spine to achieve a segmental lordosis.

**Figure 2:**
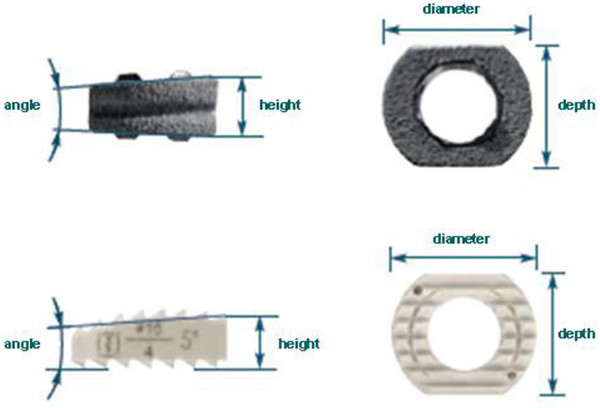
**CeSpace^®^ TTN-cage with Plasmapore^®^ coating and fixation ring (top) and CeSpace® PEEK-cage (bottom).** Note that the Peek-Cage has a slight convex shape of the upper surface, while the TTN-cage is plane.

The patients received either a CeSpace® Titan cage with Plasmapore® coating or a CeSpace® PEEK cage (B Braun Aesculap, Tuttlingen, Germany) (see Figure 2). Both cage types were applied in sizes from 4-7 mm in height with a diameter of 16 mm, depth of 13.5 mm and an angle of 5°. The choice of cage size depended mainly on the height of the adjacent intervertebral disc space and the sagittal profile. The cage was chosen to be at least 1 mm higher than the affected disc, but was not supposed to exceed a normal adjacent level disc substantially. The exact disc height of the normal adjacent level was not measured pre- or intraoperatively, but was estimated by cage trials and lateral fluoroscopy during surgery. The choice of the cage material depended on the surgeon’s personal preference.

After surgery, all patients were treated by the same protocol, which consisted of physical rest for 6 weeks and then physical therapy. A cervical collar was not applied.

### Clinical and radiological evaluation

Follow-up examinations were performed on an outpatient basis in our department. Neck and arm pain were measured using the Visual Analogue Scale (VAS); functionality was assessed with help of the Neck Disability Index (NDI). Overall clinical outcome was rated using Odom’s criteria.

Radiographic examinations included pre- and postoperative plain and functional radiography. Radiological analysis involved the measurement of various angles: Cervical lordosis was measured between C2 and C7 according to Cobb in neutral position as well as in extension and flexion. The segmental angles of the operated vertebral levels were measured in neutral position as well as in extension and flexion. Additionally, the preoperative anterior and posterior disc height of the operated and the adjacent cranial level was measured. At the last follow-up the occurrence of anterior and posterior bone bridging as well as cage subsidence (≥2 mm) [20] were assessed. Solid arthrodesis was rated according to the following accepted criteria [1,5,13,21]: The operated segment was rated as a solid arthrodesis, if movement of less than 2° was measured, and by the absence of motion between the spinous processes on lateral flexion-extension radiographs. Movement of ≥2° on flexion/extension radiographs was regarded as a pseudarthrosis [2,5].

Measurements were done on digital radiographs using integrated software to measure angles and distances up to the accuracy of 0.1° and 0.01 mm, respectively (Centricity Enterprise Web, General Electric Medical Systems, Chalfont St Giles, United Kingdom). The values were expressed as mean with standard deviation. To validate the assessed data the measurements were performed independently by two examiners. Furthermore, the measurement of the depth of the used cages (13.5 mm) in lateral radiographs served to validate the measurements.

### Statistical analysis

The statistical evaluation was performed using PASW Statistics 18, Version 18.0.0 (SPSS Inc.). Statistical analysis of ASD and gender was performed by Pearson's chi-square test. The clinical and radiological data were analysed by the Mann–Whitney-U-test, Chi-square-test and the Student's t-test. A p-value <0.05 was deemed as statistically significant.

## Results

86 of 101 eligible patients (54 men and 32 women) were evaluated. The patients’ age at time of surgery ranged from 32 to 74 years, with a mean of 54.3 years. The patients of the PEEK-group were significantly older than the patients of the TTN-group (p = 0.030). The follow-up period ranged from 12 to 60 months (mean: 28.4 months) (see Table 1).

### Surgery

ACDF was performed in 7 cases at C3/4, in 17 cases at C4/5, in 43 cases at C5/6 and in 19 cases at C6/7. The operation time ranged from 56 to 150 minutes, with a mean of 84.4 minutes. The 5 mm cage was the most frequently chosen implant in both groups. All of these parameters were evenly distributed between the groups (see Table 2). Intra- or postoperative graft dislocation did not occur.

**Table 2:** Surgical Data

**Variable**	**TTN (n = 44)**	**PEEK (n = 42)**	**p-value**
**Operated Segment**			0.077
C 3-4	1	6	
C 4-5	7	10	
C 5-6	23	20	
C 6-7	13	6	
**Operation time (min)**	91.91 ± 16.70	84.44 ± 19.99	0.133
**Cage size**			
4 mm	6	3	
5 mm	22	21	
6 mm	14	14	
7 mm	2	4	
Average Cage size	5.27 ± 0.76	5.45 ± 0.77	0.649

### Fusion

**Figure 3:**
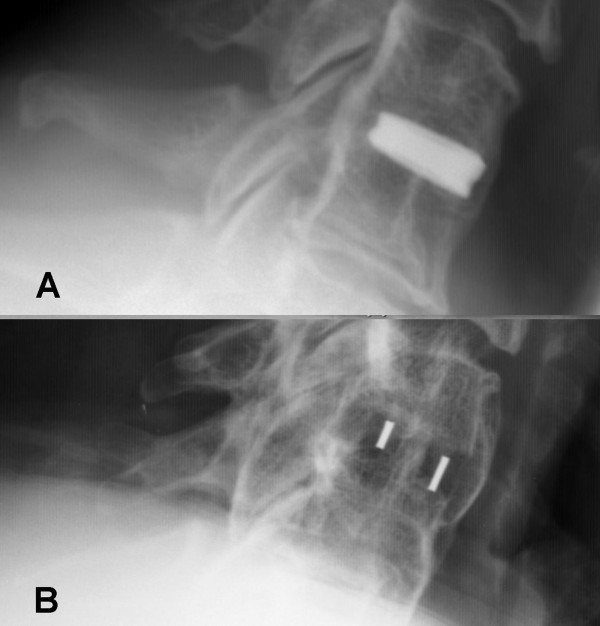
Bone formation could be seen in the TTN-group (A) and PEEK-group (B).

A solid arthdodesis was found in 93.2% of cases in the TTN-group and 88.1% of the PEEK-group (p = 0.417) according to our criteria. Bone formation was seen in 79.6% of the TTN-group, and in 61.9% of the PEEK-group (p = 0.032) (see Figure 3).

**Figure 4:**
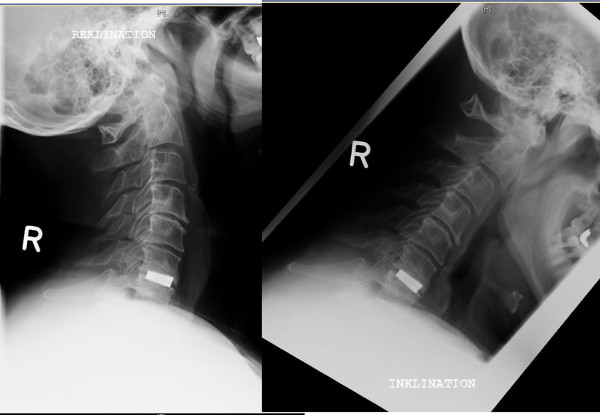
**55-years old patient with a pseudarthrosis 3 years after surgery.** The motion of the operated segment can be clearly seen by observing the movement of the spinous processes in the lateral functional x-rays.

The rate of pseudarthrosis was 6.8% in the TTN- and 11.9% in the PEEK-group, but allowed no appropriate statistical comparison due to the small number of cases (see Table 3 and Figure 4).

**Table 3:** Arthrodesis, pseudarthrosis and cage-subsidence with total number and percentage

**Variable**	**TTN (n = 44)**	**PEEK (n = 42)**	**p-value**
**Arthrodesis[n]**	41 (93.18%)	37 (88.1%)	0.417
(Segment ROM < 2°)			
**Pseudarthrosis**	3 (6.8%)	5 (11.9%)	
(Segment ROM ≥ 2°)			
**Bone Bridging**	**35 (79.55%)**	**26 (61.9%)**	0.032
Anterior	32 (72.7%)	23 (54.76%)	0.053
Posterior	28 (63.6%)	17 (40.48%)	0.031
**Cage-Subsidence**	9 (20.45%)	6 (14.28%)	0.451
Cage subsidence	3.07 ± 0.33	2.94 ± 0.64	0.601
[mm] mean ± SEM			

### Cage subsidence

**Figure 5:**
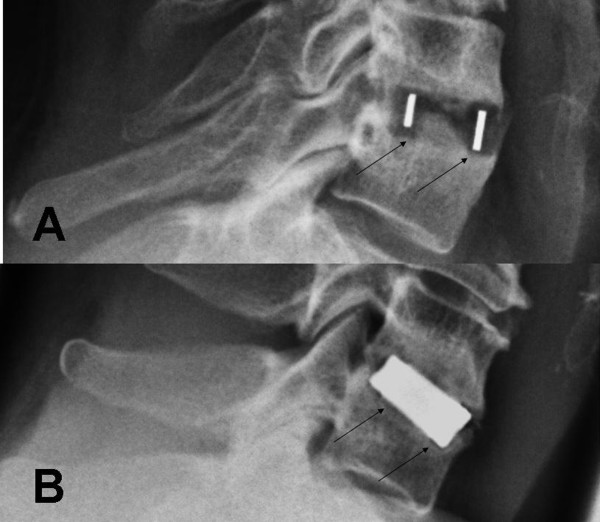
**Subsidence of a PEEK-cage into the posterior part of the inferior endplate (A) and subsidence of a TTN-cage into the anterior part of the inferior endplate (B).** A radiolucent gap can be seen in both cases around the cage (arrows).

A cage subsidence of at least 2 mm was detected in 20.5% of the TTN-group and 14.3% of the PEEK-group, but did not differ significantly between the groups (p = 0.451) (see Table 3). The amount of cage subsidence ranged from 2.02 mm to 3.88 mm and did not differ significantly between the groups as well (p = 0.601). 73.3% of cage subsidence was found in the anterior part and 26.7% in the posterior part without differences between the groups (see Table 3 and Figure 5). Cage subsidence was not affected by cage-size or disc height, ratio of preoperative disc height and cage-size, smoking behaviour or age of the patient (p > 0.117). The interobserver error was 0.11 mm assessing the amount of cage subsidence and did not affect statistical analysis.

The preoperative disc height of the operated segment in the TTN-group measured 3.71 ± 1.38 mm anterior and 3.10 ± 1.15 mm posterior. The disc height of the operated segment in the PEEK-group was 3.98 ± 1.25 mm anterior and 2.79 ± 1.14 mm posterior. Both groups did not differ (p > 0.211). The disc height of the operated level was significantly reduced compared to the adjacent cranial level in both groups (p < 0.009).

The interobserver error was 0.30 mm and 0.41 mm assessing disc height of the operated and adjacent level, respectively, and almost reached the level of statistical significance between the examiners (p > 0.07). Since the validity of disc height measurements was inferior to the measurements of the angles, the achievement and loss of correction was assessed by comparison of cervical and segmental angles.

### Segmental and cervical lordosis

A substantial correction of segmental lordosis could be observed in both groups (p = 0.0001). At the last follow-up a significant loss of correction could be found in both groups (p < 0.020). Nonetheless, compared with the preoperative segmental angle a significant lordotic angulation could be maintained at the final follow-up (p < 0.031). There was no difference between both groups.

The preoperative cervical lordosis was substantially reduced in both groups (see Table 4B). The preoperative comparison of lordotic curvature showed a decreased lordosis in the TTN-group (p = 0.05). A substantial correction of the cervical lordosis could be found in both groups after surgery (p < 0.048). In the TTN-group an overall correction of 3.3° was achieved (see Table 4A and 4B). A loss of cervical correction was observed in both groups, especially in the PEEK group (4.35°), but did not reach the level of statistical significance (p = 0.061 in the PEEK- and p = 0.294 in the TTN-group).

**Table 4:** Segmental (A) and cervical lordosis (B)

**A) Segmental Lordosis (plain [°] mean ± SEM)**			
**Variable**	**TTN (n = 44)**	**PEEK (n = 42)**	**p-value**
**Operated Segment [°]**			
Pre-OP	2.71 ± 4.68	4.43 ± 3.37	0.054
Post-OP	5.49 ± 3.85**	6.48 ± 3.67**	0.223
Final	4.06 ± 3.65**	5.51 ± 4.13**	0.075
**B) Cervical Lordosis (plain [°] mean ± SEM)**			
**Variable**	**TTN (n = 44)**	**PEEK (n = 42)**	**p-value**
**C2-7 [°]**			
Pre-OP	10.750 ± 14.44	16.31 ± 11.18	0.050
Post-OP	14.034 ± 12.44*	17.64 ± 9.96*	0.143
Final	13.140 ± 12.71	13.29 ± 7.06	0.288

4A: **significant differences (p < 0.031) comparing pre-OP with post-OP (=correction) as well as post-OP with final within the groups (=loss of correction).

4B: *significant differences (p < 0.048) comparing pre-OP with post-OP (=correction) within the groups.

The interobserver error was 0.31° and 0.27° assessing cervical and segmental lordosis, respectively, and did not affect statistical analysis.

### Clinical outcome

75% (n = 33) of the TTN-group and 64.3% (n = 27) of the PEEK-group rated an excellent or good outcome (p = 0.395). The analysis of the clinical data did not show any significant differences in the two groups as assessed by NDI (p = 0.940), VAS (p > 0.460 for arm and neck pain) and Odom’s criteria (p = 0.229) (see Table 5). Clinical outcome was not affected by cage subsidence (p > 0.211) or presence of bone formation (p > 0.410).

**Table 5:** Clinical outcome of both groups assessed by the neck disability index (NDI), visual analogue scale (VAS) and Odom’s criteria

**Variable**	**TTN (n = 44)**	**PEEK (n = 42)**	**p-value**
**Final neck VAS-pain**	33.01 ± 19.48	36.333 ± 21.28	0.460
(0–100 mm)			
**Final arm VAS**	23.70 ± 23.78	25.76 ± 26.74	0.759
(0–100 mm)			
**Final NDI**	16.886 ± 10.24	17.047 ± 9.61	0.940
**Odom’s Criteria**			0.229
Excellent	8	5	
Good	25	22	
Fair	9	12	
Poor	2	3	
Success of surgery	33 (75%)	27(64.3%)	0.395
(=excellent + good)			

## Discussion

We present a retrospective study comparing patients after ACDF with either stand-alone TTN- or PEEK-cages. No differences in clinical outcome could be found between the two groups. Radiological comparison revealed no differences regarding the rate of solid arthrodesis or cage subsidence. A significant segmental lordotic correction could be achieved by both cage-types.

### PEEK versus Titanium

The overall rate of cage subsidence was 17.4% in both of the groups. The loss of segmental correction in all of our patients at the last follow-up could be regarded as a consequence of cage subsidence as well, but it is of note that we defined a substantial subsidence as a minimum of 2 mm in lateral radiographs [20].

The incidence of cage subsidence appeared not to be affected by the differences in modulus of material elasticity as has been assumed by some authors [16,18]. Niu et al. used cages of different sizes in the compared TTN- and PEEK-groups, although the pre-operative disc height of both groups was comparable. The PEEK-group received cages not bigger than 6 mm, while the TTN-group received cages of no less than 7 mm and 8 mm in 86% of the operated segments and even received 9 mm cages in 5 operated levels [18]. The application of cages of a substantial size may result in an increased stress of the endplates and thus increased risk of subsidence [22-24]. In the cited study solely the measurement of disc height revealed the loss of correction that led to the conclusion that TTN-cages tend to a higher loss of correction. It is not conclusively discussed, why the segmental angulation did not change accordingly to the reduction of the disc height. In our study we observed that the validity of disc height measurement is inferior to the measurement of the Cobb angle of the cervical spine. Furthermore, in the mentioned study the PEEK-cages were filled with cancellous allograft bone, while the TTN-cages were filled with local bone and calcium phosphate bone extender, which may result in different radiological outcomes due to different osteogenic properties. The study of Chou and colleagues favoured PEEK over TTN, but examined only 9 patients in the PEEK-group [16]. Therefore, the statement that PEEK-cages are superior to TTN-cages in maintaining interspace height and achieving fusion is not entirely conclusive.

Both cage-types offer certain advantages in spine surgery. TTN-implants likely provide a good osseointegration [9]. Furthermore, their surface structure appears to be comparably resistent to microbial adhesion, although of course many factors affect the incidence of infection [25,26].

The PEEK’s modulus of elasticity is close to that of cortical bone and its radiolucency allows for a more accurate assessment of osseous fusion on plain radiographs. It does not compromise MRI-examinations, which is of particular interest in follow-up examinations of patients suffering from myelopathy and neoplastic diseases [27,28].

### Cage subsidence

When comparing PEEK- with TTN-cages the occurrence of TTN-cage subsidence is believed to be related to the higher modulus of elasticity [16,18], but comparison of cages of the same material reveals a multifactorial genesis of cage subsidence [20,29,30].

The patients in our PEEK-group were significantly older than in the TTN-group. This could be a random effect, but could also reflect the intuitive reaction of the surgeon towards potentially weaker bone substance in elder patients and consecutive choice of a cage-material with a more favourable modulus of elasticity. Also older patients can be expected to have weaker bone and therefore a potentially higher rate of subsidence. This phenomenon is a potential bias that possibly could have favoured the TTN group. A subsidence of TTN-cages is observed in 13 to 45% of cases in larger series [10,20]. The reported rate of PEEK-cage subsidence varies from 11 to 18% [17,24,31]. This shows that even the PEEK’s favourable modulus of elasticity does not prevent a cage subsidence. The different rate of cage subsidence in various studies dealing with synthetic cages might be also due to different criteria (1 or 2 mm) and measurement methods [10,20,24]. High resolution digital radiographs and digital measurement tools enable a more precise analysis of various conditions in the spine.

The distance of the implant from the anterior rim, a big cage size, small contact area of endplates and cage or overdistraction with subsequently increased stress of the endplates are possible explanations for a cage subsidence [10,20,22-24,32]. Our cages had an identical surface area and were placed close to the anterior rim. The ratio of cage size and disc height was no influencing factor on cage subsidence.

Bone quality, graft placement, force, shape of the implant and preparation of the endplates are further major factors that influence cage subsidence [20,29,30].

Revision surgery in case of cage subsidence without clinical symptoms was deemed unnecessary in our series. This goes conform with other studies [10,24,31].

### Cervical profile and disc height

The mean cervical lordosis of our patients measured around 13° and is comparable with patients suffering from DDD [12,33,34]. It reflects an already substantial loss of lordosis that normally is about 34° [35]. We managed to improve the cervical lordosis, but still did not achieve nearly normal alignment, which is hardly possible by a monosegmental approach. Furthermore, it is reported that patients may develop a kyphotic angulation at the levels above surgery [34] and subsequent loss of a transient lordotic overall correction. The loss of correction in our PEEK-group does not reach the level of significance, but is notable and could be explained by a disease progression and loss of muscle strength of the older patient collective.

Many authors determine the disc height to measure the achievement and loss of correction [16,36], but the shape of cervical vertebral bodies is, in contrast to the thoracal spine, not consistently rectangular. The concavity of the cervical disc space and the common presence of osteophytes in DDD can compromise the validity of the measurement. Our interobserver error determining disc height was higher than for the measurement of angles, thus we used the measurement of the Cobb angle to determine the achievement and loss of correction [11,12,33,34].

### Bone formation

The higher rate of bone formation in our TTN-group could be explained by three factors: 1.The patients were younger and could possibly muster larger osteogenic abilities. 2. The Plasmapore® coating of the TTN-cages enlarges the surface and might increase osteoconductive properties compared to the PEEK-cage. 3. Cage subsidence and subsequent exposure of cancellous bone inside the cage might promote fusion in certain cases [31].

The fusion rate of empty TTN-cages is reported to reach even 100% [13] and exceeds the fusion rate of empty PEEK-cages that is reported to be 72% [31]. One possible theory claims that fusion of empty cages may occur as the result of endplate failure and subsequent filling of a cage by fracture fragments. In this case the elastic mode of PEEK might prove to be disadvantageous for some patients according to the authors [31]. Nonetheless, it must be considered that fusion is not mandatory for a clinical success, and a loss of disc height and a potential segmental kyphosis might result from a cage subsidence. The comparatively little number of bone formation in our study may relate to the choice of diagnostic means and criteria to asses bony fusion. Assessing bone formation within the cages [31] was no criterion in our PEEK-group. This would have compromised a proper comparison with the TTN-group on lateral radiographs. Also even the PEEK-cages are not entirely radiolucent to allow a certain assessment of bone within the cages. For these reasons only the presence of anterior or posterior bone formation was rated.

The absence of segmental movement was our main criterion to rate a segment as a solid arthrodesis or pseudarthrosis. While in many studies bony union of the operated segment is regarded as the main criterion for a stable fusion [37,38], the study of functional flexion-extension radiographs and the position of the spinous processes can reveal motion nonetheless [39] or show a stable segmental status despite the lack of osseous trabeculation [33]. The absence of bony fusion can occur with absence of motion even for a long-term period, which is therefore accepted as a successful criterion for fusion in lateral radiographs [1,2,5,13,21,33]. Nonetheless, we preferred the term solid arthrodesis instead of fusion to describe the absence of motion. The comparison of our patients with and without bone formation revealed no clinical difference as well [31]. A CT-scan would have allowed a more precise statement of bony ingrowth, but was not performed routinely in our series to avoid an unnecessary radiation exposure.

### Surgery and study design

The surgeries were performed by three neurosurgeons in identical surgical technique in the same neurosurgical department. Nonetheless, this represents a potential bias of the study. The retrospective study design represents the major limitation of the study. The range of follow-up examinations provides a larger degree of interpretation compared to prospective studies with fixed follow-up appointments for all included patients. Preclinical data are limitedly available, and the only clinical outcome measurement is Odom’s criteria. The clinical comparison of patients suffering from myelopathy and radiculopathy is difficult, but we have focused on the radiological results of our study, particularly the rate of cage subsidence.

Postoperative external immobilization is often required when stand-alone devices are used. However, we did not observe any case of cage extrusion in our study population. This may be due to the surface structure of the cages, which either feature teeth (PEEK) or are coated with Plasmapore® and use a fixation ring (TTN). Some surgeons advocate rigid internal fixation and cervical immobilization postoperatively to prevent graft migration and nonunion and to enhance fusion [40,41]. The outcomes of rigid plate fixation have been equivocal to surgery without internal fixation in some studies [42-44]. In degenerative spondylosis the application of an internal fixation or a collar does not seem to influence the clinical or radiological outcome in a negative manner [45].

## Conclusions

Clinical and radiological outcomes of ACDF with TTN- or PEEK-cages do not appear to be influenced by the chosen synthetic graft. The occurrence of cage subsidence cannot be solely related to the cage material and its modulus of elasticity, which represents only one of many physical properties of a graft. Design, shape, size, surface architecture of a cage as well as bone density, endplate preparation and applied distraction during surgery need to be considered as further important factors.

Both grafts have certain advantages and should maintain their place in spine surgery. A multifactorial genesis of cage subsidence needs to be considered and further investigated, preferably in a prospective randomized study setting.

## Abbreviations

ACDF: anterior cervical discectomy and fusion; ASD: adjacent segment disease; CT: computer tomography; DDD: degenerative disc disease; MRI: magnetic resonance imaging; NDI: neck disability index; PEEK: polyetheretherketone; ROM: range of motion; SEM: standard error of the mean; TTN: titanium; VAS: visual analogue scale.

## Competing interests

Dr. Stefan Kroppenstedt is a consultant for B Braun Aesculap.

## Authors’ contributions

MC was responsible for conception, design, data analysis, writing and editing of the MS. SÖ DK was responsible for data analysis, writing and editing of the MS, DK was responsible for data analysis and editing of the MS, SK was responsible for conception, design, data analysis and editing of the MS. “All authors read and approved the final manuscript.”

## Pre-publication history

The pre-publication history for this paper can be accessed here:

http://www.biomedcentral.com/1471-2474/13/172/prepub
